# P94 - Food allergy in children - are we following NICE (National Centre for Clinical Excellence) guidelines in documentation?

**DOI:** 10.1186/2045-7022-4-S1-P149

**Published:** 2014-02-28

**Authors:** Srinivas Jyothi, Gaynor Pettit, Philip Debenham

**Affiliations:** 1 Birmingham Children’s Hospital, Birmingham, United Kingdom

## Background

Food allergy in children is increasingly diagnosed in recent years. While few hospitals in UK have a dedicated allergy service, many o these children are investigated by their general practitioners, paediatricians, nurse practitioners and other paediatric specialties. NICE has recently introduced guidelines on documentation of suspected food allergy in children (2011).

## Aim

Our aim was to compare documentation of children seen in outpatient clinic with suspected food allergy (tertiary children’s hospital without a dedicated allergy service) to NICE guidelines.

## Methods

We identified all out patient clinic letters with food allergy over one year period (April 2010- 11). After modifying the NICE audit proforma, we collected further information including the professional who referred these children, team reviewing them and management including discharge planning or referral to other specialties. We also collected information on final diagnosis and any referrals made to school/nursery and dietician.

## Results

We reviewed 50 of 100 eligible notes in detail. The children (aged between 4 months and 12 years) were referred mainly from GPs (64%). Most of these (80%) were seen in clinic either by Consultant Paediatrician or by allergy nurse. Egg, fish, nuts, fish were commonest allergens (total of 90%). 45 children (90%) had either specific IgE (80%) or skin prick tests (10%) performed. Final diagnosis was documented in 35 (70%) of these children (20- Definite documented food allergy, 4- Food aversion/refusal, 3- Not at risk, 2- CMPI, 2- Not food allergy, 1- Abdominal migraine). Discussion regarding adrenaline auto injector was documented in 15 (30%) and 5 (10%) had these prescribed. Less than 30% had dietician or school referral documented. 42% were discharged while 64% were still being followed up.

## Conclusions

We found good documentation of signs and symptoms with food, suspected allergen, allergen avoidance and advice on avoidance of suspected allergen (80-90%). Improvement in documentation could be achieved in family history of atopy, examination of atopy and growth, documenting final diagnosis, management, adrenaline auto-injector, referrals to dietician and school/nursery (40-70%).

## Recommendations

Proforma was developed based on NICE guidelines for documentation of allergy based history and examination. We are planning to develop “one stop allergy clinic” in near future.

